# A73 EVALUATING MENTAL HEALTH CONDITIONS IN YOUTH WITH INFLAMMATORY BOWEL DISEASE: A RETROSPECTIVE STUDY

**DOI:** 10.1093/jcag/gwad061.073

**Published:** 2024-02-14

**Authors:** K Beaudoin, E Mewhinney, J Lo, S Halder, K Bortolin, J Dowhaniuk, R Issenman, N Pai, M Sherlock, M Zachos, C Grant, K Beattie, K Prowse

**Affiliations:** McMaster University Faculty of Science, Hamilton, ON, Canada; McMaster University Faculty of Health Sciences, Hamilton, ON, Canada; McMaster University Faculty of Health Sciences, Hamilton, ON, Canada; McMaster Children's Hospital, Hamilton, ON, Canada; McMaster Children's Hospital, Hamilton, ON, Canada; McMaster Children's Hospital, Hamilton, ON, Canada; McMaster Children's Hospital, Hamilton, ON, Canada; McMaster Children's Hospital, Hamilton, ON, Canada; McMaster Children's Hospital, Hamilton, ON, Canada; McMaster Children's Hospital, Hamilton, ON, Canada; McMaster Children's Hospital, Hamilton, ON, Canada; McMaster Children's Hospital, Hamilton, ON, Canada; McMaster Children's Hospital, Hamilton, ON, Canada

## Abstract

**Background:**

Adolescents with chronic disease are at increased risk of psychosocial and socio-emotional challenges. During and after the COVID-19 pandemic, an increased prevalence of mental health conditions was observed in youth with chronic conditions. It is essential to understand the prevalence of mental health conditions in youth with Inflammatory Bowel Disease (IBD) to better support, advocate, and treat mental health conditions within a pediatric healthcare setting.

**Aims:**

We aimed to determine the number and proportion of patients with IBD at McMaster Children’s Hospital (MCH) whose medical charts have documentation of 1) a mental health condition (generalized anxiety disorder (GAD), social anxiety disorder (SAD), eating disorder, major depressive disorder (MDD), suicidal ideation, attention deficit disorder and other) and/or 2) medication(s) used to treat mental health conditions.

**Methods:**

Patients 12-17 years old with IBD who were treated in the pediatric Gastroenterology Clinic at MCH and had at least one appointment since June 4, 2022 were eligible. Medical records were reviewed to identify documented mental health conditions and patients’ current medications. The prevalence was then determined.

**Results:**

Of 114 patients (77 male) (mean (SD) age 15.1 (1.6) years old), 33 (29%, n=20 males) had ≥ 1 recorded mental health condition: GAD (n=27, 82%), SAD (n=1, 3%), eating disorders (n=4, 12%), MDD (n=9, 27%), suicide ideations (n=5, 15%), attention deficit disorder (n=9, 24%), and other mental health conditions (n=1, 3%). Among the 33 patients with documented mental health conditions, 19 (58%) patients were taking medications related to mental health (Table I).

**Conclusions:**

Our results estimate a 29% prevalence of mental health conditions in youth with IBD at MCH. A lack of mental health resources and screening protocols within this setting could result in an underrepresentation of adolescents with IBD and mental health comorbidities. Future studies will focus on incorporating screening methods for mental health conditions within pediatric healthcare settings to determine current barriers and accessibility to mental health supports.

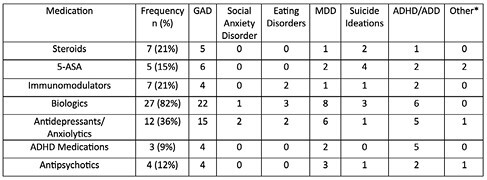

Table I. Medications in patients (n=33) with documented mental health condition

**Note:**

Medications were prevalent in patients with ampersand:003E1 mental health diagnosis, thus frequency is lower than # of documented mental health diagnoses.

*Other mental health conditions: borderline personality disorder and bipolar disorder with depression.

**Funding Agencies:**

None

